# Factors Associated with Underutilization of Maternity Health Care Cascade in Mozambique: Analysis of the 2015 National Health Survey

**DOI:** 10.3390/ijerph19137861

**Published:** 2022-06-27

**Authors:** Sérgio Chicumbe, Maria do Rosário Oliveira Martins

**Affiliations:** 1 Health System and Policy Program, Instituto Nacional de Saúde, Estrada Nacional 1, Parcela N° 3943, Vila de Marracuene 020502, Mozambique; 2 Global Health and Tropical Medicine, Instituto de Higiene e Medicina Tropical, Universidade Nova de Lisboa, Rua da Junqueira N° 100, 1349-008 Lisboa, Portugal; mrfom@ihmt.unl.pt

**Keywords:** factors, underutilization, maternity health care, Mozambique

## Abstract

Maternity health care services utilization determines maternal and neonate outcomes. Evidence about factors associated with composite non-utilization of four or more antenatal consultations and intrapartum health care services is needed in Mozambique. This study uses data from the 2015 nationwide Mozambique’s Malaria, Immunization and HIV Indicators Survey. At selected representative households, women (*n* = 2629) with child aged up to 3 years answered a standardized structured questionnaire. Adjusted binary logistic regression assessed associations between women-child pairs characteristics and non-utilization of maternity health care. Seventy five percent (95% confidence interval (CI) = 71.8–77.7%) of women missed a health care cascade step during their last pregnancy. Higher education (adjusted odds ratio (AOR) = 0.65; 95% CI = 0.46–0.91), lowest wealth (AOR = 2.1; 95% CI = 1.2–3.7), rural residency (AOR = 1.5; 95% CI = 1.1–2.2), living distant from health facility (AOR = 1.5; 95% CI = 1.1–1.9) and unknown HIV status (AOR = 1.9; 95% CI = 1.4–2.7) were factors associated with non-utilization of the maternity health care cascade. The study highlights that, by 2015, recommended maternity health care cascade utilization did not cover 7 out of 10 pregnant women in Mozambique. Unfavorable sociodemographic and economic factors increase the relative odds for women not being covered by the maternity health care cascade.

## 1. Introduction

Skilled antenatal consultation (ANC), institutional delivery (ID) and timely post-natal consultation (PNC) are cost-effective services with great impact on maternal, unborn baby and neonate survival. High coverage of maternity health care is a longstanding World Health Organization (WHO) recommendation, especially for under resourced African settings [1,2,3]. The efforts to increase health care coverage need to be sustained if trends on maternal and perinatal deaths are to be improved by 2030, as per the Sustainable Development Goals (SDG), at global regional and country levels. Global health agreements pledge to attain improved maternal and neonatal survival through services offered to the entire maternal health care cascade. The SDG pays special attention to low-income African countries. Sub-Saharan Africa remains a priority region for the goals and for maternity health care improvements. Indeed, sub-Saharan African countries still share the highest maternal and neonatal death burden, with regional figures in 2017 as high as 533 maternal deaths/100,000 live births and 27 newborn deaths/1000 live births [4].

Maternity health care coverage metrics are well established [5,6]. Thanks to global efforts and financing, several low- and middle-income countries in Africa and elsewhere benefit from demographic and health surveys which provide quality reliable population-based data to measure maternity health care coverage indicators. Demographic and health survey outputs are indeed a reference to shape informed health policy and related interventions. Health survey data were consistently collected over at least three decades in several sub-Saharan African countries, and are used to assess key maternal and child health outcomes and service coverage [7]. Remarkable progress in ANC coverage was achieved in sub-Saharan Africa by 2015 compared to 1990s levels [8]; to a lower extent, progress also occurred to ID and PNC coverage [1,8,9,10,11]. However, the predominant use of maternal, neonatal and child health survey data is for detailed analysis of maternal and child health care coverage regarding ANC, ID or PNC services. Few studies analyze composite health care cascade coverage that comprises all three of the abovementioned components. Analysis of health care cascade coverage enables improvement to the holistic assessment of progress in the provision of maternity health care as set out by national health programs [6,12].

Thereby, studies focusing on low- and middle-income countries are needed to elicit the coverage of maternity health care cascade composite indicators as a mean to monitor performance of the health care services [13]. Maternity health care cascade assessment is recommended by the World Health Organization, and its results are expected to facilitate policy makers’ systemic views of health services delivery [14]. Monitoring maternal-neonatal health care cascade coverage would likely contribute to the design of integrated health service interventions, which currently are seemingly fragmented in several settings [15,16]. 

Mozambique had shortcomings in delivering the Millennium Development Goals by 2015 and beyond [17,18,19]. Indeed, after maternal deaths steadily decrease between the 1990s and 2000s, Mozambique’s maternal and neonatal mortality rates had stagnated by 2017, with 452 maternal deaths per 100,000 live births [20] and 27 newborn deaths per 1000 live births [4,21], respectively. Furthermore, it was estimated that interventions offered through the maternity health care cascade may save an additional 3640 mothers and over 18000 children yearly in Mozambique [22]. 

Mozambican women are entitled to a maternity health care cascade that is free of charge, available, accessible and of the highest quality, and includes four or more ANC, qualified ID and PNC, as per national guidelines [23,24]. The maternity health care cascade is provided at the primary health level, mostly at health centers, in which such services are frequently run by mid-level obstetric and child health clinical practitioners. In the event of high obstetric risk or complications at ANC, ID or PNC, women are hierarchically referred to maternity health care at secondary, tertiary and quaternary levels of care (hospitals), according to severity of the risk or complication [23]. In Mozambique’s hospitals, obstetric clinical practitioners are a mix of mid-level, higher level and specialist health workers in obstetrics, neonatology and child health. At third and fourth levels hospitals, maternity and child health care teams and services are often led by obstetricians and pediatricians [25].

Apart from availability and accessibility, consistent utilization of a quality maternity health care cascade is key to decrease maternal and child mortality in countries such as Mozambique. The latest available data for Mozambique was derived from the survey conducted in 2015, which, being a population-based representative health survey, is considered the reference and most reliable measurement of maternity health service utilization. The survey captures women’s socioeconomic, demographic, health knowledge and seeking behavior covariates, which theoretically determine health care service utilization, as applied in similar studies [26,27,28,29]. 

It should not be acceptable that women exposed to one component of the maternal health care service miss other health care steps throughout the health care cascade. Gaps still exist in the evidence about the extent and determinants of women missing components of the maternal health care cascade in Mozambique. Therefore, this study aims to assess levels of, and factors associated with, maternal healthcare cascade underutilization in Mozambique. For the dependent variable, the study outputs focus on the non-utilization category rather than utilization. Although binary categories of the dependent variable mirror each other, the non-utilization category is of much interest and is more meaningful for maternal health policy discussions. 

## 2. Materials and Methods

### 2.1. Data Source

This study is based on a secondary analysis of the 2015 Mozambique national survey on HIV, malaria and immunization indicators (MZAIS 2015, locally designed as IMASIDA 2015). The survey applied women and child health questionnaires to which respondents were women of childbearing age (15–49 years old). The survey collected general household composition, assets and family members characteristics, demographics of the women and of their child/ren born since January 2010, child health, women reproductive characteristics and utilization of health services during pregnancy (antenatal, delivery and post-natal care); this latest survey primarily aimed to estimate HIV prevalence in the adult population and malaria prevalence amongst children. Specific HIV and malaria questions and biomarkers were also available, but were deemed not required for the current assessment. The data is publicly available on request through the Demographic and Health Survey Program (DHS) website—www.dhsprogram.com/data (accessed on 5 January 2019) [7]. 

### 2.2. Sample

The survey used a multistage random sampling that was representative at provinces, national and rural-urban levels. Sampling strategies and detailed survey procedures are published in the survey report [30] and in Demographics and Health Surveys program’s reference documents [6]. A 7169-household sample was included in the MZAIS 2015 survey. The survey had above 97% response rate and included 7749 women aged 15–49 years for interview on reproductive, maternal and child health. 

While the survey included women with children up to 5 years old by the time the survey was implemented, comprehensive questionnaires on reproductive health and experiences with health services, namely ANC, ID and PNC, were applied to women who gave birth to children between 1 January 2013 and the survey date [31]. Thus, this study uses a MZAIS 2015 sub-sample of 2629 Mozambican women, who had a living child born between 1 January 2013 and the interview date in 2015.

### 2.3. Control Variables

Control variables are contingent to the MZAIS 2015 questionnaires. These include sociodemographic characteristic such as age, marital status, employment, schooling, wealth index, accessibility to health facility, place of residence, health-seeking behavior and other health matters (e.g., immunization, HIV status, knowledge). 

We transformed some covariates into commonly used factors. For example, age was recorded in years, but was thereby recoded to the commonly used 5-year span categories; the family members number, and time to reach a health setting, primarily recorded as discrete numbers, were both categorized into binary variables; having access to, and use of television or radio or newspaper were composed into “access to media”; several questions on how decisions are taken within households allowed to compose “participating in household decision”; the child immunization status was adjusted to the child’s age and national immunization calendar [32], which allowed to compute a binary variable “immunization up-to-date”. 

Wealth index was computed by the data provider using household assets principal components analysis. Survey weights were also provided by the DHS program. Computation methods are published in the DHS program reference manual [6]. All other categorical variables were composed and used as routinely applied in similar studies using DHS program databases [29,33]. 

### 2.4. Stepwise (Non) Utilization Indicators 

Several stepwise computations were conducted to prepare intermediate standalone binary variables as components of the maternity health care cascade, according to the following: (i) any number of ANC for the index birth; (ii) any number of ANC with skilled health professional, being skilled professional midwife or mother and child health nurse or doctor; (iii) 4 or more ANC; (iv) 4 or more ANC provided by skilled health worker; (v) adequate ANC content, adopting similar approach from published literature [13]; the “adequate ANC content” is defined if women had at least 0.75 (3 or more) of the 5 ANC components, namely counselling on HIV vertical transmissions, measures to prevent HIV infection, how to access HIV testing, how to perform an HIV test and malaria chemoprophylaxis; (vi) institutional delivery in a health center or hospital; vii) post-childbirth consultation by the 28th day; (viii) consultation by the 28th day post birth with a skilled health worker; (ix) post-birth consultation by the 60th day and (x) post-birth consultation by 60 days post birth with a skilled worker. Appendix A, Table A2 describes outputs of the abovementioned maternity health care standalone indicators, relative frequencies and 95% confidence intervals. 

The computation of “qualified” health facility excluded the health posts. This exclusion considers the fact that health centers and hospitals are exclusively the Mozambican health facility types qualified for basic (BEmNOC) and comprehensive (CEmNOC) emergency neonatal and obstetric health care, respectively. This construct is thereby aligned to national policies [25,34]. We excluded community health workers from skilled health professionals since, by the year 2015, they were neither trained nor qualified to assist and were not allowed to classify perinatal and obstetric cases according to the, then, national policy [35]. 

**Figure 1:**
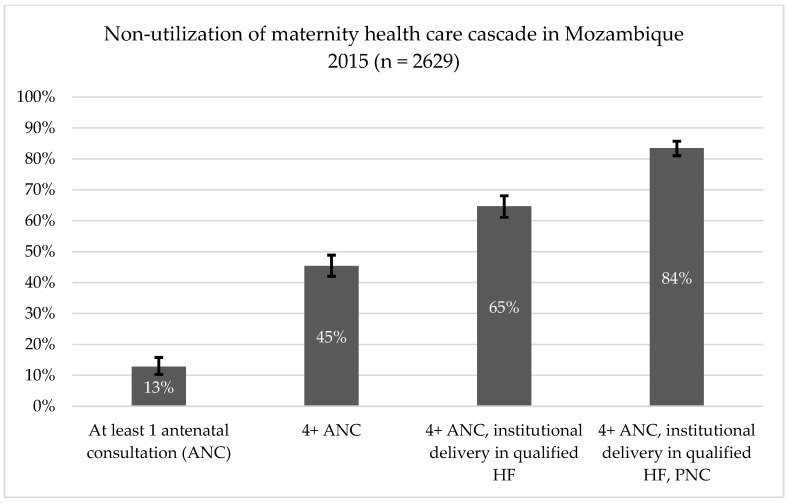
Levels of non-utilization of select maternity health care cascade components among women respondents (*n* = 2629) of the 2015 national health survey, Mozambique. Notes: ANC—ante natal consultation, HF—health facility; PNC—postpartum (post-natal) consultation.

The non-utilization the PNC cascade step was only considered in the descriptive analysis outputs shown in Figure 1 and Appendix A, Table A2. The specific PNC stand-alone indicator computation considered: (i) an intermediate PNC step, that is, whether the neonate-mother dyad had PNC within 28 days post birth and (ii) whether the neonate-mother dyad had a consultation completed within 60 days post birth. These cut-offs of the post-birth consultation periods are both of much interest for early HIV diagnosis and treatment initiation amongst HIV-exposed infants as established by national health care guidelines [3,23,34]. The computation of both PNC standalone indicators as guided by HIV program policy was deemed important given the high HIV prevalence (15%) amongst Mozambican women of childbearing age, one of the highest HIV prevalence worldwide [30,36].

### 2.5. Dependent Variable for Regression Model: ANC and ID Composite Indicator

We constructed the dependent variable, after the above explained stepwise stand-alone intermediate health care cascade indicators, based on published concepts about maternal and child health care [16]. In turn, the dependent variable used for the regression analysis is thereby, sequential, and conditional to non-utilization of 1, 2, 3, 4 or more ANC with skilled professional, or delivery out of a qualified health center or hospital. Thus, the dependent variable was computed as a binary composite variable comprising non-utilization of, any ANC or four or more ANC or ID in a qualified health facility. Because non-utilization of the maternity health care cascade is the outcome of interest, for analysis purposes, non-utilization is coded as success taking the value 1, while utilization is coded as 0. 

### 2.6. Statistical Analysis

We first conducted univariate analysis to describe the women’s sociodemographic and economic characteristics and presented weighted proportions and 95% confidence intervals. Second, we described non-utilization proportions for each step of the maternity health care cascade and its respective 95% confidence intervals. Third, we cross-tabulated the “maternity health care cascade” non-utilization from ANC up to ID against sociodemographic, reproductive and health-seeking behavioral factors, and presented crude odds ratios (COR) and COR 95% confidence intervals (CI). In step four, we used binary logistic regression to compute adjusted odds ratios (AOR) and respective 95% confidence interval to identify factors with definitive significant associations with the dependent variable. Confidence intervals not crossing the null value (null = 1) indicated statistically significant associations. Computation, transformations, recodification and analysis were performed using the Statistical Package for Social Sciences (SPSS version 24.0 (New York, NY, USA)). All analyses were weighted and adjusted for the survey complex sampling.

## 3. Results

Proportions of respondents by sociodemographic category, reproductive characteristics and/or prophylaxis and health-seeking behavior are described in Table 1. Of note, is that 42.5% of respondents were from Mozambique’s central region and 74.9% were from rural areas. Of all respondents, 66.7% were aged between 15 and 30 years, 28.5% were illiterate and 44.9% engaged in activities for income. Catholic and Islamic women corresponded to 29.2% and 19.9% of the sample, respectively.

The proportion of women residing in households with a means of transportation, including at least having a bike, was 46.6%; piped water source was available for 30.9%, and 8.4% interviewees resided in households with improved toilet facilities. The proportion of respondents taking part in domestic decisions was 9.5% and 64.0% resided more than 30 min walking distance from the closest health facility.

Concerning gestation and preventative health-seeking behavior for the index child, results show that 30.7% of interviewed women had 5 or more pregnancies and 79.2% wanted the index child pregnancy at the time she became pregnant. Although only 14.7% knew the immunization calendar, 72.9% had the index child completely vaccinated for what was expected for the child’s age; 72.2% were still breastfeeding and the status of child’s exposure to HIV was known in 68.4% of cases. In Appendix A, Table A1, percent distribution of respondents’ sociodemographic and health characteristics is shown disaggregated by non-users and users of the maternal health care cascade up to institutional delivery at a qualified health facility.

Figure 1 shows selected stepwise non-utilization of the maternity health care cascade relative frequencies. The figure shows that 45.4% of women had not reached 4 or more antenatal consultations (ANC) as per guidelines [1,2,3]; 65% did not reach a combination of four or more ANC and childbirth at qualified health centers or hospitals; and 84% did reach the combination of having had four or more ANC, qualified institutional delivery and post-natal consultation (PNC) services with a qualified professional within 60 days after childbirth. The full list of standalone stepwise non-utilization indicators is shown in the Appendix A, Table A2. Considering the cascade composed of four or more ANC, birth in a health setting and PNC within 60 days after delivery and excluding the requisite of a qualified health settings and health professionals, results show that 74.9% (95% CI = 71.8–77.7%) of respondents were non-users of the maternal health care cascade (Appendix A, Table A2). 

Table 2 shows factors associated with non-utilization of the maternity health care cascade up to ID. Women residing in rural areas had 1.5 times higher relative odds (95% CI = 1.05–2.17) of non-utilization of the health care cascade. Women residing in Cabo Delgado, Tete, Sofala, Inhambane and Maputo provinces, as compared to Maputo city, had 60% lower relative odds, and women from Nampula had 50% lower relative odds of not using the health care cascade; mother’s educational level and wealth quintile were also associated with higher odds ratios of non-utilization of the health services. Indeed, women with high schooling had 35% lower relative odds (AOR = 0.65; 95% CI = 0.46–0.91) of not utilizing the health care cascade as compared with women with primary education. Mothers residing in households with the lowest wealth index had higher health care service cascade non-utilization relative odds (AOR = 2.06; 95% CI = 1.15–3.71) when compared to women from “wealthier” households; similarly higher maternity health care cascade non-utilization odds ratio was verified for mothers in other lower wealth indexes (AOR = 1.78; 95% CI = 1.04–3.04). 

Result show women excluded from domestic decisions had higher relative odds of missing steps of the health care cascade (AOR = 1.45; 95% CI = 1.03–2.03). Women residing a long distance from a health setting (defined as more than 30 min walking to reach the facility) had 1.5 times higher relative odds of being lost from the health care cascade (AOR = 1.46; 95% CI = 1.11–1.91); women who were unaware of the status of child’s exposure to HIV have almost 2 times higher odds ratio of non-utilization of the health care cascade (AOR = 1.93; 95% CI = 1.40–2.67).

Although shown to be associated in the step of bivariate analysis, women’s age, marital cohabitation duration, past travel, number of pregnancies and child’s immunization status were not associated with non-utilization of the health care cascade in the respective adjusted odds ratio (AOR) 95% confidence interval (Table 2).

## 4. Discussion

Our study aimed to analyze levels and determinants of non-utilization of ANC and ID in Mozambique using the most recent and nationally representative population data. The study also describes levels of non-utilization of standalone and composite steps of maternity health care, from ANC, ID and PNC. High coverage of the maternal health care cascade is a core long-running programmatic strategy, which is ultimately known to impact women and child morbimortality in low-income countries and elsewhere. Indeed, the maternity health care cascade is not only cost-efficacious and largely proven to require low-technology, but is also highly scalable to reach those most in need of the health care [3]. 

Despite increased ANC coverage over at least three decades, as shown by the MZAIS 2015 report [30], about a quarter of pregnant women still do not utilize the recommended four or more antenatal consultations. About 75% of women do not comprehensively use all steps of maternity health care services despite accessing the health system through at least one ANC. This indicates critical missed opportunities to provide peripartum health care as is programmatically intended, which, in turn, would determine better outcomes for mother-unborn baby pair, and neonates. 

As shown in our study, majority of women from the studied population are young, lowly educated, have already had multi-gestations, reside in rural areas and in socio-economically poor households. A third of women did not know whether her child was HIV exposed, and a third had a child with missed immunizations relative to the national guidelines. Although the survey reference period were three years before 2015, these results point to persistent challenges to achieve improved mother and child health, not only by the end of the Millennium Development Goals (MDG) in 2015, but also likely in the current Sustainable Development Goals (SDG) era. This suggests that better maternal and child health care cascade coverage must be urgently implemented [18]. 

Overall, our result points to the multifaceted, though amenable, sociodemographic and economic challenges that women-baby pairs are still facing in Mozambique; the identified socioeconomic factors associated with non-utilization of the health care cascade likely share the same developmental roots and come under the responsibility of sectors beyond the Ministry of Health. Other studies [37,38,39] had previously shown the sociodemographic and economic status of women as determinants of their ability to attend health care in Mozambique. Some other studies from sub-Saharan Africa had also pointed to similar factors in the early 2000s [28,40,41,42]. 

Our results showed that the socioeconomic conditions in which women live were still unfavourable (water, fuel, sanitation, distance to health facilities) by 2015. After substantial investments in the health sector in Mozambique across decades [43,44,45], either by the mother and child health program and by development partners, it is a worrisome finding that 75% of eligible women were not covered by a second, third or fourth ANC or were not delivering at a qualified maternity service.

In Mozambique, user fees are not applicable for pregnant women and to pairs of a mother and an under-5-year-old child. To upscale the maternal and child health care cascade utilization, Mozambique’s strategy across the MDG era (years 2000–2015) focused on increasing the availability, quality and proximity of health services to communities [22,43,46], as well as revitalizing linkages to care through the community health workers referral of pregnant, child birthing, puerperal women and neonates [35]. Contrary to several countries, Mozambique had not employed financial incentives at the demand side for maternal and child health coverage. In Asia, financial incentive schemes were used to protect costs incurred by women when accessing health services. Financial intervention increased in the specific contexts of the service’s utilization, especially institutional deliveries [47,48,49]. However, in the very same Asian context, there are calls for financial intervention adjustments so that equity distribution of demand-side financial incentives is achieved [48,50,51], a feature Mozambique needs not to apply in order to increase maternal and child health care cascade utilization.

Our analysis focused on women who entered the ANC at least once and were not covered by any of the consecutive maternal health care steps. As shown in other publications, perceived health care quality may limit consecutive service utilization [13,40,41,52,53]. We have not explicitly assessed perceptions about quality antenatal care. However, besides being a topic for further studies, there are few reports from Mozambique suggesting that health care quality is an issue. For example, Mozambican women do, indeed, wait hours at health facilities for only a short time—for an average 9 min consultation [54,55]. Thus, a possible mechanism underlying health care cascade under coverage may include the opportunity costs for socioeconomically disadvantaged women to comply with the entire health care cascade. Of note, is that our study population was shown to be largely socioeconomically disadvantaged women. It is reasonable to assume that demand side economic factors may be interplaying for women adhering to recommended frequent consultation visits, as required by the maternity health care policy in Mozambique. 

Our study showed about 91% of women did not participate in decisions within their households. The decision to seek care, for example, place where to give birth, should be jointly taken by women and family members; previous studies have shown husbands’ positive attitudes toward health, amongst factors allowing women to give birth at health facilities [39,56,57,58]. Birth preparations, preventative measures uptake and responsiveness toward incident complications during the pregnancy, peripartum and postpartum, are influenced by family decision-making dynamics [59,60]. Thereby, empowering women to be entitled to participate in decisions within their households will likely increase service utilization and ultimately improve maternal and child health outcomes in Mozambique and elsewhere.

Maternal-child health care cascade non-utilization was shown to be associated with a long distance to reach health facilities. As indicated in other studies from the same region as Mozambique, and elsewhere [38,61,62,63,64,65], our findings confirms the need to have community health services supporting maternity health care interventions and linkages. Strengthened outreach activities for ANC and PNC counting on skilled health workers are likely scalable options. This would require strengthening manpower for maternal and child health at the primary and community level, which in Mozambique, is still lacking [39,66,67]. For deliveries, improving referral systems, ready maternities and promoting health-seeking behavior amongst pregnant women may contribute to improve utilization, and, through these, positively impact the persisting high maternal-neonatal morbimortality [63,68,69]. The above suggested interventions are already included in global and local strategies to advance maternal-child health care in low-income countries such as Mozambique [70,71,72]. 

It has been demonstrated that ANC utilization is an important institutional delivery predictor [28,73,74], as are also factors the past solved peripartum complications or positive obstetric experience [75,76,77]. Conversely, it is a novelty in our study that antenatal consultations, instead of being determinants or factors, are part of the study dependent variable. Furthermore, to the best of our knowledge, the entire maternal health care cascade analysis is novel in Mozambique.

Exposure to mass media, by proxy, means to reach and incentivize women to utilize health services through health promotion activities. Our descriptive analysis showed that 76.5% of women had no meaningful access to radio or television. Media receivers, amongst other household assets, are also inputs to the wealth quintile index, and we found that the lower wealth quintile is associated with non-utilization of the maternal health care cascade. Others, as we did, also found access (distance) to health services, rurality and wealth of women as factors determining institutional deliveries [38,48,52,56,63,69,77,78,79,80]. 

Differing from other studies [74,81,82,83,84], women’s age in our study was not associated with non-utilization of maternal-child health services. Nevertheless, often the youngest women, characteristically dominant in our study population, do require novel approaches for linking their specific needs to health setting responsiveness. Contextual shaping interventions for young women population, therefore, remain a key feature in scaling up service utilization [12,85,86].

One study limitation derives from ours being cross-sectional and questioning for past events. Questioning past events may face recall bias. However, the survey methodology employed is validated over many decades. Furthermore, the questionnaire for obstetric events further restricted the sample toward women with children born up to 3 years previously, rather than commonly practice of 5 years previously. After this restriction, the sample size was still large (above 2600 respondents), thereby the study power was maintained. We excluded traditional birth attendants (TBA), community health workers (CHW) and health posts from skilled ANC, ID and PNC qualified health care attendant, which can be seen by others as a limitation. However, we believe that these restrictions strengthened the definition of skilled attendants necessary for quality and high standards of maternity health care in Mozambique, since the TBA and CHW were decisively not trained nor entitled to provide ANC, ID or PNC attendance by 2015. They could, however, only refer cases in need of ANC, ID or PNC; health posts are not accredited, nor commodity or infrastructurally ready for the mentioned maternity health care cascade. We believe, thereby, that despite the mentioned potential limitations, results are still valid to inform policies and maternal and child health care practice and policy discussions.

## 5. Conclusions

In the beginning of Sustainable Development Goals era, still about 7 out of 10 pregnant women missed the maternal-child health care cascade in Mozambique. Reaching higher coverage of ANC, ID and PNC attendance as a continuum still remains a core public health goal. Persisting multilevel sociodemographic and economic factors, which are dependent on multisectoral actions, are associated with non-utilization of the cost-efficacious maternal-child essential and basic health care packages. 

## Figures and Tables

**Table 1:** Women’s sociodemographic and health characteristics.

Variables	% [95% CI]
Region	
North	37.4 [35.6–39.2]
Center	42.5 [40.7–44.4]
South	20.1 [18.5–21.6]
Residence	
Urban	25.1 [23.5–26.8]
Rural	74.9 [73.2–76.5]
Other sociodemographic	
Age 15–19 (years)	17.2 [15.8–18.7]
Age 20–24 (years)	28.7 [27.0–30.4]
Age 25–29 (years)	20.8 [19.3–22.4]
Age 30–34 (years)	14.8 [13.5–16.2]
Age 35+ (years)	18.4 [16.9–19.8]
Cohabitation 0–4 years	35.3 [33.5–37.0]
Cohabitation 5+ years	64.7 [63.0–66.5]
Illiterate	28.5 [26.8–30.2]
Primary education	55.4 [53.5–57.2]
Secondary education	16.2 [14.8–17.5]
Catholic	29.1 [27.4–30.8]
Islamic	19.9 [18.4–21.4]
Other	50.9 [49.0–52.8]
Activity with income	44.9 [43.0–46.8]
Own transport vehicle	46.6 [44.7–48.4]
Number of family members is 4+	83.7 [82.3–85.1]
Travel in past 12 months	20.6 [19.0–22.1]
Watch/listen/read media	23.5 [21.9–25.1]
Cooking energy is coal or wood	99.7 [99.1–100]
Piped water source	30.9 [29.2–32.7]
Improved toilet	8.4 [7.4–9.5]
Take part in household decisions	9.5 [8.4–10.6]
Health related	
Used mosquito net for child	57.1 [55.2–58.9]
Gesta 1	22.1 [20.5–23.6]
Gesta 2	19.5 [18–20.9]
Gesta 3	15.3 [14–16.7]
Gesta 4	12.4 [11.2–13.6]
Gesta 5+	30.7 [29–32.4]
Wanted child	79.2 [77.7–80.8]
Want more children	59.0 [57.1–60.8]
Child is male	48.8 [46.9–50.7]
Currently breastfeeding	72.2 [70.5–73.8]
Time to primary health care facility >30′	64.0 [62.1–65.9]
Know EPI calendar	14.7 [13.3–16.0]
Immunization is up-to-date	72.9 [71.3–74.6]
Known child HIV exposure status	68.4 [66.6–70.1]
Total sample *n*	2629

EPI—expanded immunization program; HIV—human immunodeficiency virus; CI—confidence interval.

**Table 2:** Factors associated with non-utilization of maternity health care cascade.

Dependent Variable: Non-Utilization of 4+ ANC and Institutional Delivery				
	Explanatory Variables	Refs.	Crude Odds Ratio [95% CI]	Adjusted Odds Ratio [95% CI]
Age (years)	20–24	[15,16,17,18,19]	1.13 [0.82–1.56]	1.03 [0.69–1.52]
	25–29	[15,16,17,18,19]	1.09 [0.78–1.52]	0.75 [0.47–1.18]
	30–34	[15,16,17,18,19]	1.02 [0.71–1.46]	0.68 [0.38–1.20]
	35–39	[15,16,17,18,19]	1.69 [1.19–2.40]	0.94 [0.50–1.78]
Province	Niassa	Maputo city	3.02 [1.47–6.23]	0.69 [0.31–1.54]
	Cabo Delgado	Maputo city	1.74 [0.96–3.16]	0.42 [0.23–0.78] *
	Nampula	Maputo city	2.3 [1.32–4.0]	0.51 [0.27–0.97] *
	Zambézia	Maputo city	3.16 [1.69–5.92]	0.52 [0.26–1.03]
	Tete	Maputo city	2.28 [1.1–4.72]	0.43 [0.19–0.97] *
	Manica	Maputo city	3.63 [1.93–6.83]	1.13 [0.63–2.03]
	Sofala	Maputo city	1.19 [0.7–2.03]	0.35 [0.20–0.61] *
	Inhambane	Maputo city	0.87 [0.53–1.45]	0.35 [0.20–0.64] *
	Gaza	Maputo city	1.55 [0.97–2.47]	0.72 [0.42–1.23]
	Maputo	Maputo city	0.51 [0.32–0.83]	0.35 [0.21–0.58] *
Residence	Rural	Urban	3.28 [2.41–4.46]	1.51 [1.05–2.17] *
Education	No education	Primary	1.64 [1.21–2.23]	1.22 [0.88–1.70]
	Secondary	Primary	0.32 [0.24–0.43]	0.65 [0.46–0.91] *
Wealth index	Poorest	Richest	6.7 [4.37–10.26]	2.06 [1.15–3.71] *
	Poorer	Richest	5.09 [3.44–7.51]	1.78 [1.04–3.04] *
	Middle	Richest	3.22 [2.17–4.8]	1.29 [0.79–2.11]
	Richer	Richest	1.76 [1.33–2.33]	1.12 [0.80–1.57]
Religion	Islamic	Catholic	1.17 [0.76–1.78]	[-]
	Other	Catholic	1.04 [0.75–1.44]	[-]
Family members	3	4+	1.09 [0.77–1.54]	[-]
Cohabitation (years)	5+	0–4	1.56 [1.26–1.92]	1.19 [0.84–1.69]
Part of household decisions	No	Yes	1.92 [1.39–2.64]	1.45 [1.03–2.03] *
Travelled	No	Yes	1.68 [1.29–2.19]	1.17 [0.89–1.54]
Gesta	2	1	1.27 [0.9–1.78]	1.37 [0.84–2.22]
	3	1	1.38 [1.01–1.9]	1.46 [0.91–2.34]
	4	1	1.54 [1.03–2.29]	1.43 [0.82–2.49]
	5+	1	1.71 [1.26–2.34]	1.22 [0.65–2.31]
Wanted child	Wanted later	Wanted	0.74 [0.56–0.96]	[-]
	Did not want	Wanted	1.16 [0.71–1.91]	[-]
Want more children	No	Yes	1.07 [0.84–1.36]	[-]
	Unsure	Yes	1.24 [0.75–2.03]	[-]
Child sex	Female	Male	1.03 [0.83–1.27]	[-]
Time to health facility	>30 min	≤30 min	2.0 [1.51–2.63]	1.46 [1.11–1.91] *
Immunization up-to-date	No	Yes	2.0 [1.45–2.76]	1.27 [0.91–1.77]
Child HIV exposure status	Unknown	Known	3.02 [2.21–4.13]	1.93 [1.40–2.67] *

* *p* < 0.05 and association different from null; CI = confidence interval; HIV = human immunodeficiency virus; [-] = no estimates of Adjusted Odds Ratio and respective CI since the bivariate Crude Odds Ratio CI crosses the null value 1.

## Data Availability

Data request should be directed to Demographic and Health Survey Program (DHS) through the website—www.dhsprogram.com/data (accessed on 5 January 2019).

## Appendix A

**Table A1:** Respondents sociodemographic and health characteristics disaggregated by nonusers and users of antenatal consultations and institutional delivery cascade, Mozambique, 2015.

	Nonusers	Users
Variables	Estimate (%) and [95% Confidence Interval]	
Province		
Niassa	7.6 [6.4–8.9] *	4.5 [3.2–5.8]
Cabo Delgado	7.8 [6.6–9.1] *	12.0 [10.0–14.1]
Nampula	24.7 [22.7–26.7] *	15.9 [13.6–18.2]
Zambezia	18.2 [16.4–20] *	8.8 [7.0–10.6]
Tete	9.8 [8.4–11.2]	7.5 [5.8–9.2]
Manica	9.8 [8.4–11.2] *	6.7 [5.1–8.3]
Sofala	8.8 [7.5–10.2]	11.9 [9.8–13.9]
Inhambane	4.1 [3.2–5.0] *	7.6 [5.9–9.3]
Gaza	4.9 [3.9–5.9] *	12.2 [10.2–14.3]
Maputo	2.0 [1.3–2.6] *	7.1 [5.4–8.7]
Maputo city	2.2 [1.5–2.9] *	5.7 [4.3–7.2]
Region		
North	40.1 [37.9–42.4] *	32.4 [29.5–35.4]
Center	46.7 [44.4–49] *	34.9 [31.9–37.9]
South	13.2 [11.6–14.8] *	32.6 [29.7–35.6]
Residence zone		
Urban	16.5 [14.8–18.2] *	40.9 [37.8–44]
Rural	83.5 [81.8–85.2] *	59.1 [56.0–62.2]
Other sociodemographic		
Age 15–19 (years)	15.9 [14.2–17.6]	19.8 [17.3–22.3]
Age 20–24 (years)	28.2 [26.1–30.3]	29.7 [26.8–32.6]
Age 25–29 (years)	21 [19.1–22.9]	20.5 [17.9–23.0]
Age 30–34 (years)	14.7 [13.1–16.4]	15.0 [12.8–17.3]
Age 35+ (years)	20.2 [18.3–22] *	15.0 [12.8–17.3]
Cohabitation 0–4 years	30.7 [28.5–32.8] *	43.6 [40.5–46.7]
Cohabitation 5+ years	69.3 [67.2–71.5] *	56.4 [53.3–59.5]
Illiterate	33.8 [31.6–36] *	18.7 [16.3–21.2]
Primary education	57.3 [55.0–59.6]	51.9 [48.7–55.0]
Secondary education	8.9 [7.6–10.3] *	29.4 [26.5–32.3]
Catholic	30.1 [28.0–32.3]	27.3 [24.5–30.1]
Islamic	20.9 [19.0–22.8]	18.0 [15.6–20.5]
Other	48.8 [46.5–51.2] *	54.6 [51.5–57.7]
Activity with income	45.3 [43.0–47.6]	44.2 [41.1–47.3]
Own transport vehicle	49.7 [47.4–52.1] *	40.7 [37.6–43.8]
Family members 4+	83.5 [81.8–85.3]	84.0 [81.7–86.3]
Travel in previous 12 months	17.3 [15.5–19.1] *	26.5 [23.7–29.3]
Watch/listen/read media	16.1 [14.4–17.8] *	37.1 [34.0–40.1]
Cooking energy coal or wood	100 [100] *	98.1 [96.8–99.4]
Piped water source	23.1 [21.1–25.1] *	45.3 [42.1–48.4]
Improved toilet	4.4 [3.4–5.3] *	15.9 [13.6–18.2]
Household decisions	7.7 [6.5–9.0] *	12.6 [10.5–14.7]
Health related		
Used mosquito net for child	54.8 [52.5–57.2] *	61.2 [58.1–64.2]
Gesta 1	18.8 [17.0–20.6] *	28.1 [25.2–30.9]
Gesta 2	19.7 [17.9–21.6]	18.9 [16.5–21.4]
Gesta 3	15.3 [13.6–17.0]	15.4 [13.2–17.7]
Gesta 4	12.8 [11.3–14.4]	11.7 [9.6–13.7]
Gesta 5+	33.4 [31.2–35.6] *	25.9 [23.1–28.6]
Wanted child	81.6 [79.8–83.4] *	75.0 [72.2–77.7]
Want more children	58.9 [56.6–61.2]	59.1 [56.0–62.2]
Child is male	48.6 [46.3–50.9]	49.2 [46.0–52.4]
Currently breastfeeding	74.4 [72.3–76.4] *	68.1 [65.2–71.1]
Time to primary health care facility >30′	70.9 [68.7–73.2] *	52.3 [49.2–55.5]
Know EPI calendar	13.6 [12.0–15.2]	16.6 [14.2–18.9]
Immunization up-to-date	67.1 [64.9–69.2] *	83.6 [81.3–86.0]
Known child HIV exposure status	61.1 [58.8–63.4] *	81.6 [79.2–84.1]
Total *n*	1584	1045

* Estimates of participant characteristics percent distribution per nonuser and user group are different from null; EPI—expanded program of immunization; HIV—human immunodeficiency virus.

**Table A2:** Maternal health care cascade non-utilization indicators, Mozambique health survey, 2015.

Non–Utilization of	%	95% CI
Any antenatal consultation (ANC)	12.8%	10.3–15.8%
4+ ANC	45.4%	42.1–48.9%
Any ANC with adequate package	49.2%	45.8–52.7%
4+ ANC with adequate package	64.8%	61.9–67.7%
Institutional delivery	30.1%	26.5–34.0%
Institutional delivery in qualified health facility (HF)	43.2%	39.2–47.4%
Post–natal consultation (PNC/PPC) within 28th day, skilled attendant	72.5%	69.6–75.2%
PNC within 60th day, skilled attendant	48.2%	44.5–51.9%
Any ANC, institutional delivery	35.7%	31.8–39.8%
4+ ANC, institutional delivery	57.3%	53.8–60.7%
4+ ANC, institutional delivery in qualified HF *	64.7%	61.1–68.1%
4+ ANC, institutional delivery qualified HF and PNC	85.8%	83.5–87.9%
4+ ANC, institutional delivery qualified HF, PNC skilled worker	83.5%	81.0–85.7%
4+ ANC, institutional delivery qualified HF, PNC 28th day with skilled worker	85.8%	83.5–87.9%
4+ ANC and institutional delivery in qualified HF and PNC within 60th day with health worker	74.9%	71.8–77.7%
Total Sample	2629	

* Indicator used as dependent variable for multivariable regression analysis; CI = confidence interval; ANC = antenatal care; PNC = post-natal consultation; PPC = post-partum consultation; HF = health facility.

## References

[1] World Health Organisation (WHO) (2018). WHO Recommendations on Intrapartum Care for a Positive Childbirth Experience.

[2] World Health Organization (2016). WHO Recommendations on Antenatal Care for a Positive Pregnancy Experience.

[3] World Health Organization (2013). WHO Recommendations on Postnatal Care of the Mother and Newborn.

[4] UNIGME (2019). Trends in Maternal Mortality 2000 to 2017: Estimates by WHO, UNICEF, UNFPA, World Bank Group and the United Nations Population Division.

[5] Ashton T. (2015). Measuring Health System Performance: A New Approach to Accountability and Quality Improvement in New Zealand. Health Policy.

[6] Croft T.N., Marshall A.M.J., Allen C.K. (2018). Guide to DHS Statistics.

[7] Demographic and Health Program The DHS Program. https://dhsprogram.com/.

[8] Oestergaard M.Z., Inoue M., Yoshida S., Mahanani W.R., Gore F.M., Cousens S., Lawn J.E., Mathers C.D., on behalf of the United Nations Inter-agency Group for Child Mortality Estimation and the Child Health Epidemiology Reference Group (2011). Neonatal Mortality Levels for 193 Countries in 2009 with Trends since 1990: A Systematic Analysis of Progress, Projections, and Priorities. PLoS Med..

[9] Blencowe H., Cousens S., Jassir F.B., Say L., Chou D., Mathers C., Hogan D., Shiekh S., Qureshi Z.U., You D. (2016). National, Regional, and Worldwide Estimates of Stillbirth Rates in 2015, with Trends from 2000: A Systematic Analysis. Lancet Glob. Health.

[10] Cousens S., Blencowe H., Stanton C., Chou D., Ahmed S., Steinhardt L. (2011). National, Regional, and Worldwide Estimates of Stillbirth Rates in 2009 with Trends since 1995: A Systematic Analysis. Lancet.

[11] Duysburgh E., Zhang W.H., Ye M., Williams A., Massawe S., Sie A., Williams J., Mpembeni R., Loukanova S., Temmerman M. (2013). Quality of Antenatal and Childbirth Care in Selected Rural Health Facilities in Burkina Faso, Ghana and Tanzania: Similar Finding. Trop. Med. Int. Health.

[12] (2015). Every Woman, Every Child, Every Adolescent: Achievements and Prospects: The Final Report of the Independent Expert Review Group on Information and Accountability for Women’s and Children’s Health.

[13] Servan-Mori E., Contreras-Loya D., Gomez-Dantes O., Nigenda G., Sosa-Rubi S.G., Lozano R. (2017). Use of Performance Metrics for the Measurement of Universal Coverage for Maternal Care in Mexico. Health Policy Plan..

[14] Bailey P., Lobis S., Fortney J., Maine D., Joseph L., Family Health International (Organization), Mailman School of Public Health, UNICEF, United Nations Population Fund, World Health Organization (2009). Monitoring Emergency Obstetric Care: A Handbook.

[15] Bucagu M., Kagubare J.M., Basinga P., Ngabo F., Timmons B.K., Lee A.C. (2012). Impact of Health Systems Strengthening on Coverage of Maternal Health Services in Rwanda, 2000-2010: A Systematic Review. Reprod. Health Matters.

[16] Bustreo F., Requejo J.H., Merialdi M., Presern C., Songane F. (2012). From Safe Motherhood, Newborn, and Child Survival Partnerships to the Continuum of Care and Accountability: Moving Fast Forward to 2015. Int. J. Gynecol. Obstet..

[17] Bryce J., Daelmans B., Dwivedi A., Fauveau V., Lawn J.E., Mason E., Newby H., Shankar A., Starrs A., Wardlaw T. (2008). Countdown to 2015 for Maternal, Newborn, and Child Survival: The 2008 Report on Tracking Coverage of Interventions. Lancet.

[18] Victora C., Requejo J., Boerma T., Amouzou A., Bhutta Z.A., Black R.E., Chopra M. (2016). Countdown to 2030 for Reproductive, Maternal, Newborn, Child, and Adolescent Health and Nutrition. Lancet Glob. Health.

[19] (2017). World Health Statistics 2017: Monitoring Health for the SDGs, Sustainable Development Goals.

[20] Instituto Nacional de Estatistica (2019). Resultados Definitivos Do IV Censo Geral Da População e Habitação 2017.

[21] UNICEF UNICEF DATA—Child Statistics. https://data.unicef.org/.

[22] Júnior J.M., Cane R.M., Gonçalves M.P., Sambo J., Konikoff J., Fernandes Q., Ngale K., Roberton T. (2019). Projecting the Lives Saved by Continuing the Historical Scale-up of Child and Maternal Health Interventions in Mozambique until 2030. J. Glob. Health.

[23] Ministério da Saúde (2014). Fluxogramas de Atendimento para os Serviços de Saúde Reprodutiva, Materna e Neonatal.

[24] Ministério da Saúde (2006). Politica Nacional de Saúde Neonatal e Infantil em Moçambique.

[25] Ministério da Saúde (2002). Diploma Ministerial 127/2002-Caracterização Técnica, Enunciado de Funções Especificas, Critérios e Mecanismos Para Classificação das Instituições do Serviço Nacional de Saúde.

[26] Shibre G., Zegeye B., Idriss-Wheeler D., Yaya S. (2021). Factors Affecting the Utilization of Antenatal Care Services among Women in Guinea: A Population-Based Study. Fam. Pract..

[27] Adewuyi E.O., Zhao Y., Auta A., Lamichhane R. (2017). Prevalence and Factors Associated with Non-Utilization of Healthcare Facility for Childbirth in Rural and Urban Nigeria: Analysis of a National Population-Based Survey. Scand. J. Public Health.

[28] Bishanga D.R., Drake M., Kim Y.-M., Mwanamsangu A.H., Makuwani A.M., Zoungrana J., Lemwayi R., Rijken M.J., Stekelenburg J. (2018). Factors Associated with Institutional Delivery: Findings from a Cross-Sectional Study in Mara and Kagera Regions in Tanzania. PLoS ONE.

[29] Bwalya B.B., Mulenga M.C., Mulenga J.N. (2017). Factors Associated with Postnatal Care for Newborns in Zambia: Analysis of the 2013-14 Zambia Demographic and Health Survey. BMC Pregnancy Childbirth.

[30] Instituto Nacional de Saúde (INS), Instituto Nacional de Estatistica (INE), ICF Macro (2015). Mozambique AIDS Indicators Survey (MZAIS) 2015.

[31] Ministerio de Saude (MISAU) (2015). Mozambique Demographic Health Survey AIS 2015.

[32] Ministério da Saúde (2011). Manual de Formação Para Técnicos de Medicina Preventiva e Saúde Do Meio, Programa Nacional Alargado de Vacinação.

[33] Yaya S., Uthman O.A., Bishwajit G., Ekholuenetale M. (2019). Maternal Health Care Service Utilization in Post-War Liberia: Analysis of Nationally Representative Cross-Sectional Household Surveys. BMC Public Health.

[34] Ministério da Saúde (2011). Manual Técnico Sobre Assistência ao Parto, ao Recém-Nascido e às Principais Complicações Obstétricas e Neonatais.

[35] Ministério da Saúde (MISAU) (2011). Programa de Revitalizacao Dos Agentes Polivalentes Elementares.

[36] Instituto Nacional de Saúde (INS), Instituto Nacional de Estatistica (INE), ICF Macro (2009). Mozambique AIDS Indicators Survey (MZAIS) 2009.

[37] Cole C.B., Pacca J., Mehl A., Tomasulo A., van der Veken L., Viola A., Ridde V. (2018). Toward Communities as Systems: A Sequential Mixed Methods Study to Understand Factors Enabling Implementation of a Skilled Birth Attendance Intervention in Nampula Province, Mozambique. Reprod. Health.

[38] Makanga P.T., Schuurman N., Sacoor C., Boene H.E., Vilanculo F., Vidler M., Magee L., von Dadelszen P., Sevene E., Munguambe K. (2017). Seasonal Variation in Geographical Access to Maternal Health Services in Regions of Southern Mozambique. Int. J. Health Geogr..

[39] Munguambe K., Boene H., Vidler M., Bique C., Sawchuck D., Firoz T., Makanga P.T., Qureshi R., Macete E., Menéndez C. (2016). Barriers and Facilitators to Health Care Seeking Behaviours in Pregnancy in Rural Communities of Southern Mozambique. Reprod. Health.

[40] Abubakar S., Adamu D., Hamza R., Galadima J.B. (2017). Determinants of Home Delivery among Women Attending Antenatal Care in Bagwai Town, Kano Nigeria. Afr. J. Reprod. Health.

[41] Agha S., Williams E. (2016). Quality of Antenatal Care and Household Wealth as Determinants of Institutional Delivery in Pakistan: Results of a Cross-Sectional Household Survey. Reprod. Health.

[42] Akibu M., Tsegaye W., Megersa T., Nurgi S. (2018). Prevalence and Determinants of Complete Postnatal Care Service Utilization in Northern Shoa, Ethiopia. J. Pregnancy.

[43] Ministério da Saúde (MISAU) (2014). Plano Estratégico Do Sector Da Saúde 2014–2019 [2025].

[44] WHO (2004). Reproductive Health Strategy to Accelerate Progress towards the Attainment of International Development Goals and Targets.

[45] World Health Organisation (WHO) (2015). Saving Lives Protecting Futures: Progress Report on the Global Strategy for Women’s and Children’s Health 2010–2015.

[46] Ministério da Saúde (2008). Roteiro Para Aceleracao Da Reducao Da Mortalidade Materna e Neonatal Em Mocambique 2008.

[47] Carvalho N., Thacker N., Gupta S.S., Salomon J.A. (2014). More Evidence on the Impact of India’s Conditional Cash Transfer Program, Janani Suraksha Yojana: Quasi-Experimental Evaluation of the Effects on Childhood Immunization and Other Reproductive and Child Health Outcomes. PLoS ONE.

[48] Gupta A., Fledderjohann J., Reddy H., Raman V.R., Stuckler D., Vellakkal S. (2018). Barriers and Prospects of India’s Conditional Cash Transfer Program to Promote Institutional Delivery Care: A Qualitative Analysis of the Supply-Side Perspectives. BMC Health Serv. Res..

[49] Lim S.S., Dandona L., Hoisington J.A., James S.L., Hogan M.C., Gakidou E. (2010). India’s Janani Suraksha Yojana, a Conditional Cash Transfer Programme to Increase Births in Health Facilities: An Impact Evaluation. Lancet.

[50] Sidney K., Tolhurst R., Jehan K., Diwan V., De Costa A. (2016). “The Money Is Important but All Women Anyway Go to Hospital for Childbirth Nowadays”—A Qualitative Exploration of Why Women Participate in a Conditional Cash Transfer Program to Promote Institutional Deliveries in Madhya Pradesh, India. BMC Pregnancy Childbirth.

[51] Silan V., Kant S., Archana S., Misra P., Rizwan S. (2014). Determinants of Underutilisation of Free Delivery Services in an Area with High Institutional Delivery Rate: A Qualitative Study. N. Am. J. Med. Sci..

[52] Dahiru T., Oche O.M. (2015). Determinants of Antenatal Care, Institutional Delivery and Postnatal Care Services Utilization in Nigeria. Pan Afr. Med. J..

[53] Exavery A., Kante A.M., Njozi M., Tani K., Doctor H.V., Hingora A., Phillips J.F. (2014). Access to Institutional Delivery Care and Reasons for Home Delivery in Three Districts of Tanzania. Int. J. Equity Health.

[54] Gong E., Dula J., Alberto C., de Albuquerque A., Steenland M., Fernandes Q., Cuco R.M., Sequeira S., Chicumbe S., Gudo E.S. (2019). Client Experiences with Antenatal Care Waiting Times in Southern Mozambique. BMC Health Serv. Res..

[55] Wagenaar B.H., Gimbel S., Hoek R., Pfeiffer J., Michel C., Cuembelo F., Quembo T., Afonso P., Gloyd S., Lambdin B.H. (2016). Wait and Consult Times for Primary Healthcare Services in Central Mozambique: A Time-Motion Study. Glob. Health Action.

[56] Dadi L.S., Berhane M., Ahmed Y., Gudina E.K., Berhanu T., Kim K.H., Getnet M., Abera M. (2019). Maternal and Newborn Health Services Utilization in Jimma Zone, Southwest Ethiopia: A Community Based Cross-Sectional Study. BMC Pregnancy Childbirth.

[57] Kaba M., Bulto T., Tafesse Z., Lingerh W., Ali I. (2016). Sociocultural Determinants of Home Delivery in Ethiopia: A Qualitative Study. Int. J. Womens Health.

[58] Ntenda P.A.M. (2019). Women’s Status within the Household as a Determinant of Institutional Delivery in Malawi. Midwifery.

[59] Belda S.S., Gebremariam M.B. (2016). Birth Preparedness, Complication Readiness and Other Determinants of Place of Delivery among Mothers in Goba District, Bale Zone, South East Ethiopia. BMC Pregnancy Childbirth.

[60] Rosado C., Callaghan-Koru J.A., Estifanos A.S., Sheferaw E., Shay T., de Graft-Johnson J., Rawlins B., Gibson H., Baqui A.H., Nonyane B.A.S. (2019). Effect of Birth Preparedness on Institutional Delivery in Semiurban Ethiopia: A Cross-Sectional Study. Ann. Glob. Health.

[61] Faierman M.L., Anderson J.E., Assane A., Bendix P., Vaz F., Rose J.A., Funzamo C., Bickler S.W., Noormahomed E.V. (2015). Surgical Patients Travel Longer Distances than Non-Surgical Patients to Receive Care at a Rural Hospital in Mozambique. Int. Health.

[62] Hanson C., Cox J., Mbaruku G., Manzi F., Gabrysch S., Schellenberg D., Tanner M., Ronsmans C., Schellenberg J. (2015). Maternal Mortality and Distance to Facility-Based Obstetric Care in Rural Southern Tanzania: A Secondary Analysis of Cross-Sectional Census Data in 226,000 Households. Lancet Glob. Health.

[63] Jain A.K., Sathar Z.A., ul Haque M. (2015). The Constraints of Distance and Poverty on Institutional Deliveries in Pakistan: Evidence from Georeference-Linked Data. Stud. Fam. Plann..

[64] Kruk M.E., Mbaruku G., McCord C.W., Moran M., Rockers P.C., Galea S. (2009). Bypassing Primary Care Facilities for Childbirth: A Population-Based Study in Rural Tanzania. Health Policy Plan.

[65] Yao J., Agadjanian V. (2018). Bypassing Health Facilities in Rural Mozambique: Spatial, Institutional, and Individual Determinants. BMC Health Serv. Res..

[66] Boene H., Vidler M., Augusto O., Sidat M., Macete E., Menéndez C., Sawchuck D., Qureshi R., von Dadelszen P., Munguambe K. (2016). Community Health Worker Knowledge and Management of Pre-Eclampsia in Southern Mozambique. Reprod. Health.

[67] Khowaja A.R., Qureshi R.N., Sawchuck D., Oladapo O.T., Adetoro O.O., Orenuga E.A., Bellad M., Mallapur A., Charantimath U., CLIP Working Group (2016). The Feasibility of Community Level Interventions for Pre-Eclampsia in South Asia and Sub-Saharan Africa: A Mixed-Methods Design. Reprod. Health.

[68] Afari H., Hirschhorn L.R., Michaelis A., Barker P., Sodzi-Tettey S. (2014). Quality Improvement in Emergency Obstetric Referrals: Qualitative Study of Provider Perspectives in Assin North District, Ghana. BMJ Open.

[69] Chavane L.A., Bailey P., Loquiha O., Dgedge M., Aerts M., Temmerman M. (2018). Maternal Death and Delays in Accessing Emergency Obstetric Care in Mozambique. BMC Pregnancy Childbirth.

[70] Augusto O., Keyes E.E., Madede T., Abacassamo F., de la Corte P., Chilundo B., Bailey P.E. (2018). Progress in Mozambique: Changes in the Availability, Use, and Quality of Emergency Obstetric and Newborn Care between 2007 and 2012. PLoS ONE.

[71] Chongo L., Amade N., Chavane L., da Luz Vaz M., David E., Dos Anjos M., Ricca J., Arscott-Mills S., Rosen H., Drake M. (2013). Quality and Humanization of Care Assessment (QHCA).

[72] Jhpiego (2015). Save the Children Maternal and Child Health Integrated Program Final Report 2015.

[73] Fekadu A., Yitayal M., Alemayehu G.A., Abebe S.M., Ayele T.A., Tariku A., Andargie G., Teshome D.F., Gelaye K.A. (2019). Frequent Antenatal Care Visits Increase Institutional Delivery at Dabat Health and Demographic Surveillance System Site, Northwest Ethiopia. J. Pregnancy.

[74] Séraphin M.N., Ngnie-Teta I., Ayoya M.A., Khan M.R., Striley C.W., Boldon E., Mamadoultaibou A., Saint-Fleur J.E., Koo L., Clermont M. (2015). Determinants of Institutional Delivery among Women of Childbearing Age in Rural Haiti. Matern. Child Health J..

[75] Asefa A., Gebremedhin S., Messele T., Letamo Y., Shibru E., Alano A., Morgan A., Kermode M. (2019). Mismatch between Antenatal Care Attendance and Institutional Delivery in South Ethiopia: A Multilevel Analysis. BMJ Open.

[76] Asefa A., Bekele D. (2015). Status of Respectful and Non-Abusive Care during Facility-Based Childbirth in a Hospital and Health Centers in Addis Ababa, Ethiopia. Reprod. Health.

[77] Shah R., Rehfuess E.A., Maskey M.K., Fischer R., Bhandari P.B., Delius M. (2015). Factors Affecting Institutional Delivery in Rural Chitwan District of Nepal: A Community-Based Cross-Sectional Study. BMC Pregnancy Childbirth.

[78] Llop-Gironés A., Julià M., Chicumbe S., Dulá J., Odallah A.A.P., Alvarez F., Zahinos I., Mazive E., Benach J. (2018). Inequalities in the Access to and Quality of Healthcare in Mozambique: Evidence from the Household Budget Survey. Int. J. Qual. Health Care.

[79] Mekonnen Y., Mekonnen A. (2003). Factors Influencing the Use of Maternal Healthcare Services in Ethiopia. J. Health Popul. Nutr..

[80] Zelalem Ayele D., Belayihun B., Teji K., Admassu Ayana D. (2014). Factors Affecting Utilization of Maternal Health Care Services in Kombolcha District, Eastern Hararghe Zone, Oromia Regional State, Eastern Ethiopia. Int. Sch. Res. Not..

[81] Acharya P., Adhikari T.B., Neupane D., Thapa K., Bhandari P.M. (2017). Correlates of Institutional Deliveries among Teenage and Non-Teenage Mothers in Nepal. PLoS ONE.

[82] Bayu H., Fisseha G., Mulat A., Yitayih G., Wolday M. (2015). Missed Opportunities for Institutional Delivery and Associated Factors among Urban Resident Pregnant Women in South Tigray Zone, Ethiopia: A Community-Based Follow-up Study. Glob. Health Action.

[83] Gayawan E. (2014). Spatial Analysis of Choice of Place of Delivery in Nigeria. Sex. Reprod. Healthc. Off. J. Swed. Assoc. Midwives.

[84] Uddin J., Pulok M.H., Johnson R.B., Rana J., Baker E. (2019). Association between Child Marriage and Institutional Delivery Care Services Use in Bangladesh: Intersections between Education and Place of Residence. Public Health.

[85] Banke-Thomas O.E., Banke-Thomas A.O., Ameh C.A. (2017). Factors Influencing Utilisation of Maternal Health Services by Adolescent Mothers in Low-and Middle-Income Countries: A Systematic Review. BMC Pregnancy Childbirth.

[86] Granja A.C., Machungo F., Gomes A., Bergström S. (2001). Adolescent Maternal Mortality in Mozambique. J. Adolesc. Health.

